# A Rare Case of Retroperitoneal Leiomyoma

**DOI:** 10.1155/2012/425280

**Published:** 2012-07-31

**Authors:** Rajiv Mahendru, Geetinder Gaba, Shweta Yadav, Gurmeet Gaba, Chinky Gupta

**Affiliations:** ^1^Department of Obstetrics and Gynecology, Bhagat Phool Singh Government Medical College, Khanpur Kalan 131303, Sonepat, India; ^2^Department of Obstetrics and Gynecology, Maharishi Markandeshwar Institute of Medical Sciences, Ambala 134003, India; ^3^Department of Obstetrics and Gynecology, Gaba Hospital, Yamunanagar 135001, India; ^4^Department of Pathology, Maharishi Markandeshwar Institute of Medical Sciences, Ambala 134003, India

## Abstract

*Introduction*. Leiomyoma uteri is one of the most common benign conditions for which women undergo hysterectomy every year. Fibroids found retroperitoneally are a rare entity, especially, primary retroperitoneal fibroid. *Case Presentation*. We report a case of 42-year-old para 1 who presented to our hospital with recurring retention of urine, lower abdominal and pelvic pain, and dyspareunia . Provisional diagnosis on the basis of examination and imaging was large subserosal fibroid with mild right-sided hydroureteronephrosis, due to pressure effect of the fibroid. Abdominal hysterectomy was done for the patient, and intraoperatively, a bulky uterus was found with multiple small fibroids on anterior and posterior walls, and a large fibroid approx. 10 × 8 cm was found arising from the posterior surface at the level of internal os retroperitoneally, which was confirmed by histopathology as leiomyoma. *Conclusion*. Retroperitoneal fibroids are rare neoplasms and treatment is surgical removal. Preoperative imaging can only give provisional diagnosis and can be misguiding. Final diagnosis of retroperitoneal fibroid can be made only intraoperatively.

## 1. Introduction

Leiomyomata is a common benign conditions arising from smooth muscle cells. Approximately 20%–30% of women older than 35 years have uterine leiomyomata that are manifested clinically [1, 2]. Retroperitoneal leiomyomata has a rare occurrence and has recently been recognized as distinctive lesions with similar histological features as uterine leiomyoma. Poliquin et al. [3] studied features of about 100 cases of retroperitoneal leiomyoma. This rare entity is usually misdiagnosed preoperatively even with diagnostic imaging. We report a rare case of a large retroperitoneal fibroid.

## 2. Case Presentation

A 42-year-old female patient, presented to our gynaecology outpatient department with complaints of lower abdominal pelvic pain and backache, dyspareunia, and difficulty in passing urine for the last 6 months. She had suprapubic pain with constant backache and heaviness in the perineum. Patient took medication from local practitioners for symptomatic relief. She presented to us when already catheterised with self-retaining Foley's catheter from outside for retention of urine for one day. There was history of similar episodes of retention of urine 4-5 times in the last 6 months for which she had to be catheterised once and again for about 5–7 days each time. The catheter was removed after toning of bladder.

**Figure 1:**
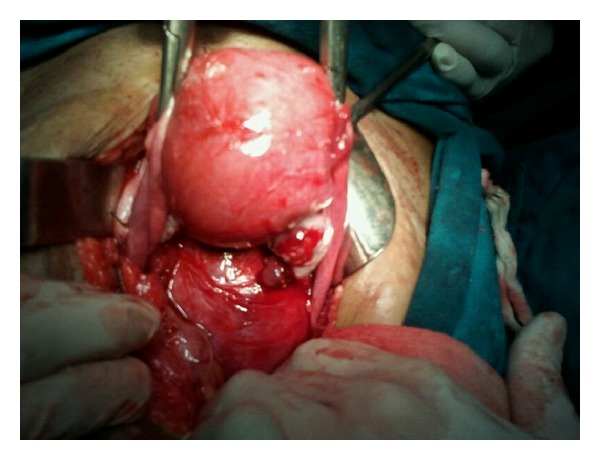
Uterus and retroperitoneal leiomyoma on initial appearance.

**Figure 2:**
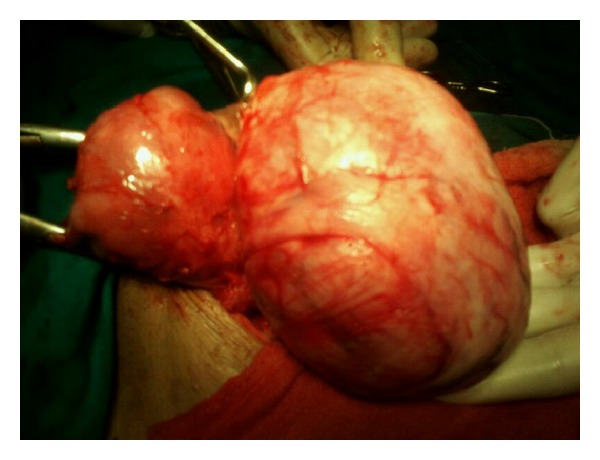
Hysterectomy and excision of retroperitoneal leiomyoma in progress.

Patient was para one with one live issue 20 years old conceived spontaneously and delivered by normal vaginal delivery and had three spontaneous first trimester abortions, thereafter. Menarche was attained at the age of 13 years. Patient had irregular menstrual cycles occurring at every 15–20 days with bleeding lasting for 4-5 days; the amount of blood flow was normal. There was no previous illness or surgical intervention. Patient was a vegetarian, nonsmoker, nonalcoholic with normal bowel habits. She was moderately built and nourished. General condition of the patient was stable with mild pallor. Vitals were normal. Abdominal examination revealed a firm mass of 16-week size arising from the pelvis deviated more towards the left side. The mass had smooth surface with restricted mobility. On pelvic examination, cervix was hitched below the pubic symphysis, bleeding was present through the cervical os. Foleys catheter was in situ. Uterus was acutely retroverted; a mass of about 16-week size, irregular, firm in consistency was felt through posterior and the left lateral fornix. The uterus could not be differentiated from the mass. Base line investigations and renal function tests of the patient were normal. Ultrasonography revealed a large uterine fibroid of size 14 × 9 × 8 cm, which was subserous with normal bilateral adnexae. Right-sided hydroureteronephrosis was also reported for which IVP was done showing right-sided ureteric obliteration with bladder neck compression. Considering the above clinical findings and investigations, provisional diagnosis of subserous fibroid was made and abdominal hysterectomy was planned. A vertical midline infraumbilical incision was employed. Intraoperatively, uterus was found to be just bulky. A large retroperitoneal firm mass of size 10 cm × 8 cm was found arising from the posterior surface at the level of internal os (Figure 1). Total abdominal hysterectomy was carried out along with removal of the large retroperitoneal mass (Figure 2). Complete haemostasis was achieved and abdomen closure was done in layers. Cut section of the hysterectomy specimen showed a few small intramural fibroids on the anterior and the posterior wall of the uterus. The retroperitoneal mass depicted whorled appearance with no apparent degeneration or calcification. The hysterectomy specimen and the retroperitoneal mass were sent for histopathological examination.

**Figure 3:**
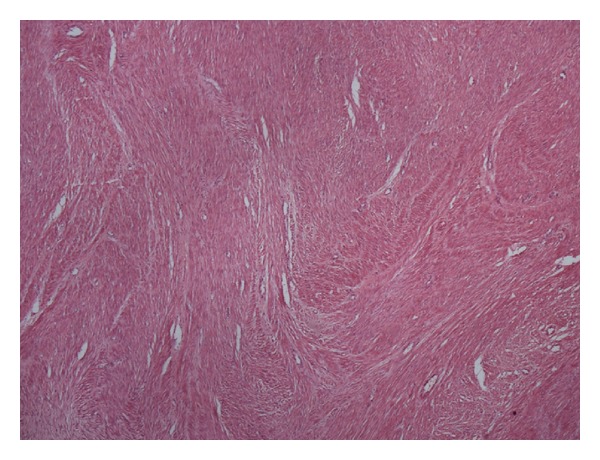
Histopathological picture.

The postoperative period of the patient was uneventful. The stitch removal was done on the seventh postoperative day. The stitch line was healthy with good healing. Histopathological examination confirmed the smooth muscle cell origin of the retroperitoneal mass consistent with diagnosis of retroperitoneal leiomyoma (Figure 3).

## 3. Discussion

Uterine fibroids are the most common benign solid pelvic tumours in women [4] and are present in about 80% of all hysterectomy specimens [5]. The most common sites of fibroid are uterus and GIT; however, it can originate wherever smooth muscle cells exist [4, 6, 7]. The extrauterine leiomyoma presentations mentioned in the literature are benign metastasizing leiomyoma, disseminated peritoneal leiomyomatosis, intravenous leiomyomatosis, parasitic leiomyomata, and retroperitoneal growth, and the unusual sites of origin include the vulva, ovaries, urinary bladder, and urethra. Some rare locations are sinonasal cavities, orbits, kidneys, and skin [8]. Retroperitoneal fibroids are rarely diagnosed preoperatively even with imaging techniques like USG, CT, and MRI, and in most of the case reports of retroperitoneal leiomyomata, preoperative diagnosis of the growth was made to be either subserous fibroid, ovarian malignancy, or fibroma [9, 10]. Kho and Nezhat [11] studied 12 cases of parasitic leiomyomata distinct from uterus, which included two retroperitoneal fibroids. They suggested iatrogenic parasitic myoma formation as the cause of retroperitoneal fibroid. With regard to their pathological origin, it is unclear whether these retroperitoneal lesions represent metastatic or synchronous primary lesions and whether they arise from the hormonally sensitive smooth muscle elements [12] or from the embryonal remnants of mullerian or wolffian duct [13]. Kang et al. [14] suggested primary multifocal origin of retroperitoneal fibroids. Common symptoms of retroperitoneal fibroids include abdominal discomfort, fatigue, backache, dyspareunia, and urinary and bowel complaints. More than 40% of patients affected by this retroperitoneal condition have a concurrent uterine leiomyoma or a remote history of hysterectomy for treatment of a uterine leiomyoma [3]. The reporting of such cases dates back to 1903, when Lewers [15] reported a case of retroperitoneal fibroid of thirteen and a half pounds. Surgical removal of the mass is the main stay of treatment, which can be by laparotomy or laparoscopic removal. Kondo et al. [16] reported a case of retroperitoneal fibroid where resection was done by laparoscopic approach. Abdominal hysterectomy along with the resection depends on the age of the patient, her symptomatology, and associated uterine myoma. The prognosis of the patients with retroperitoneal fibroid is good.

## 4. Conclusion

Retroperitoneal leiomyomata is a rare neoplasm and thorough imaging, though, helpful in operative planning may also miss the exact site of these fibroids. Final diagnosis is made intraoperatively on direct visualisation. Complete excision with or without abdominal hysterectomy is the treatment of retroperitoneal fibroids.
